# Enterorenal Fistula as an Unusual Complication from Ureteroscopic Lithotripsy: A Case Report

**DOI:** 10.1089/cren.2018.0102

**Published:** 2019-05-30

**Authors:** Sabah Akbani, J. Stuart Wolf, E. Charles Osterberg

**Affiliations:** Department of Surgery and Perioperative Care, Division of Urology, Dell Medical School at the University of Texas at Austin, Austin, Texas.

**Keywords:** enterorenal fistula, ureteroscopic lithotripsy, urosepsis

## Abstract

***Background:*** This case highlights an enterorenal fistula as a rare complication from ureteroscopic lithotripsy.

***Case Presentation:*** A 56-year-old woman with significant obesity, decompensated cirrhotic and ascitic liver disease, hypertension, type 2 diabetes mellitus, and nephrolithiasis treated with five prior ureteroscopic lithotripsies for a partial left staghorn stone presented to the emergency department (ED) with worsening left flank pain and sepsis. A CT scan of the abdomen and pelvis with contrast showed a large left perinephric hematoma. She underwent drain placement and during fluoroscopic imaging, there was a fistula from the left subcapsular hematoma/abscess to the proximal descending colon. The patient wished to proceed with a surgical course involving nephrectomy with hemicolectomy despite extensive counseling regarding her high mortality risk. However, because of worsening nutritional status as well as several other high-risk comorbidities, a shared decision was made with the patient to postpone the procedure. The patient was discharged to a skilled nursing facility for nutritional optimization and prehabilitation; however, she continued to decline with recurrent sepsis and cirrhosis-related complications and unfortunately passed away.

***Conclusion:*** A subscapular hematoma evolving into a perinephric abscess is a rare but known complication of ureteroscopic lithotripsy; however, this patient developed an enterorenal fistula that has yet to be reported after repeated ureteroscopy.

## Background

Existing literature cites perinephric and subscapular hematomas as rare complications after ureteroscopic lithotripsy with incidences of 0.3% and 0.5%, respectively.^1^ Perinephric hematomas often resolve spontaneously and do not require surgical intervention. In an even more rare instance, the renal hematoma can become infected leading to a fulminant perinephric abscess. Known risk factors for this complication include obstructive upper urinary tract infection, immunosuppression such as with diabetes, neuropathic bladder, and patients with polycystic disease on hemodialysis.^2^

Enterorenal fistula formation is also a rare occurrence. A recent literature review identified 158 cases of enterorenal fistula, of which 59% were renocolic in nature.^3^ In a review of pyeloduodenal fistulas, Rodney et al. determined that they were more likely to form with urinary obstruction. Pyelonephritis, perinephritis, and pyonephrosis were found to be risk factors in 83% of the 28 cases, of which 65% involved a renal calculi.^4^ Additional individual cases have been reported in literature involving chronic renal inflammation and recurrent urinary infections in patients with xanthogranulomatous pyelonephritis.^5–8^ Others describe iatrogenic enterorenal fistulas as complications from ablation for renal cell carcinoma or from percutaneous nephrolithotomy.^9,10^ In all of these cases, renal inflammation and obstruction, with or without infection, played a key role in the formation of a fistula tract.

We present a unique case of a patient with a history of five ureteroscopic lithotripsies complicated by an infected perinephric hematoma who was found to have a spontaneous enterorenal fistula.

## Case Presentation

The patient is a 56-year-old woman who has a history of morbid obesity (body mass index: 49.23 kg/m^2^), decompensated nonalcoholic steatohepatitis with cirrhosis and refractory ascites requiring three prior paracenteses each draining 4.5–8.5 L (Model for End Stage Liver Disease score: 31), hypertension, type 2 diabetes mellitus, and nephrolithiasis treated with five prior ureteroscopic laser lithotripsies.

She presented to the emergency department (ED) at our institution with fever and left flank pain. Before presentation, she had suffered from calcium phosphate kidney stones for several years and was taking daily potassium citrate. Ten months prior, she had been found to have a partial staghorn calculus in the left kidney, measuring 3.8 × 2.2 cm and causing incomplete obstruction. Her right kidney was unremarkable. Extracorporeal shockwave lithotripsy was not recommended. The patient opted against a left percutaneous nephrolithotomy, and instead underwent five ureteroscopic laser lithotripsies at another institution for the next 4 months.

Each of the first three sessions took place roughly 1 week apart, after which she developed Steinstrasse extending from the distal to proximal ureter. As a result, she was taken back for a fourth session within 3 weeks of her previous ureteroscopy, and then a fifth session 2 months later. During these procedures, the patient had vancomycin-resistant enterococcus in her urine by culture for which she received ciprofloxacin continuously. Owing to the size of her stone burden, each procedure was lengthy (>2 hours), and ureteral access sheaths were used to facilitate drainage. A stent was placed at the conclusion of each ureteroscopy.

After her last ureteroscopy, now 5 months before the index presentation to the ED, she developed worsening flank pain, which prompted a CT scan that revealed a 19.4 × 13.4 × 15.8 cm subscapular hematoma and a 10.4 × 3.3 × 13.5 cm perinephric hematoma along the lateral aspect of the left kidney. A ureteral stent was replaced and per report frank pus effluxed from the stent. A nuclear medicine lasix renal imaging study was attempted to assess left kidney blood flow, which showed left renal activity of 3% but was inconclusive secondary to the patient's morbid obesity and the large hematoma. At this point, she was offered a simple nephrectomy owing to a loss in renal parenchyma with residual stones despite her five ureteroscopies. Two weeks before her planned simple nephrectomy, which had been delayed several months because of her comorbidities, her index presentation to our institution with fever and flank pain occurred.

**FIG. 1.**
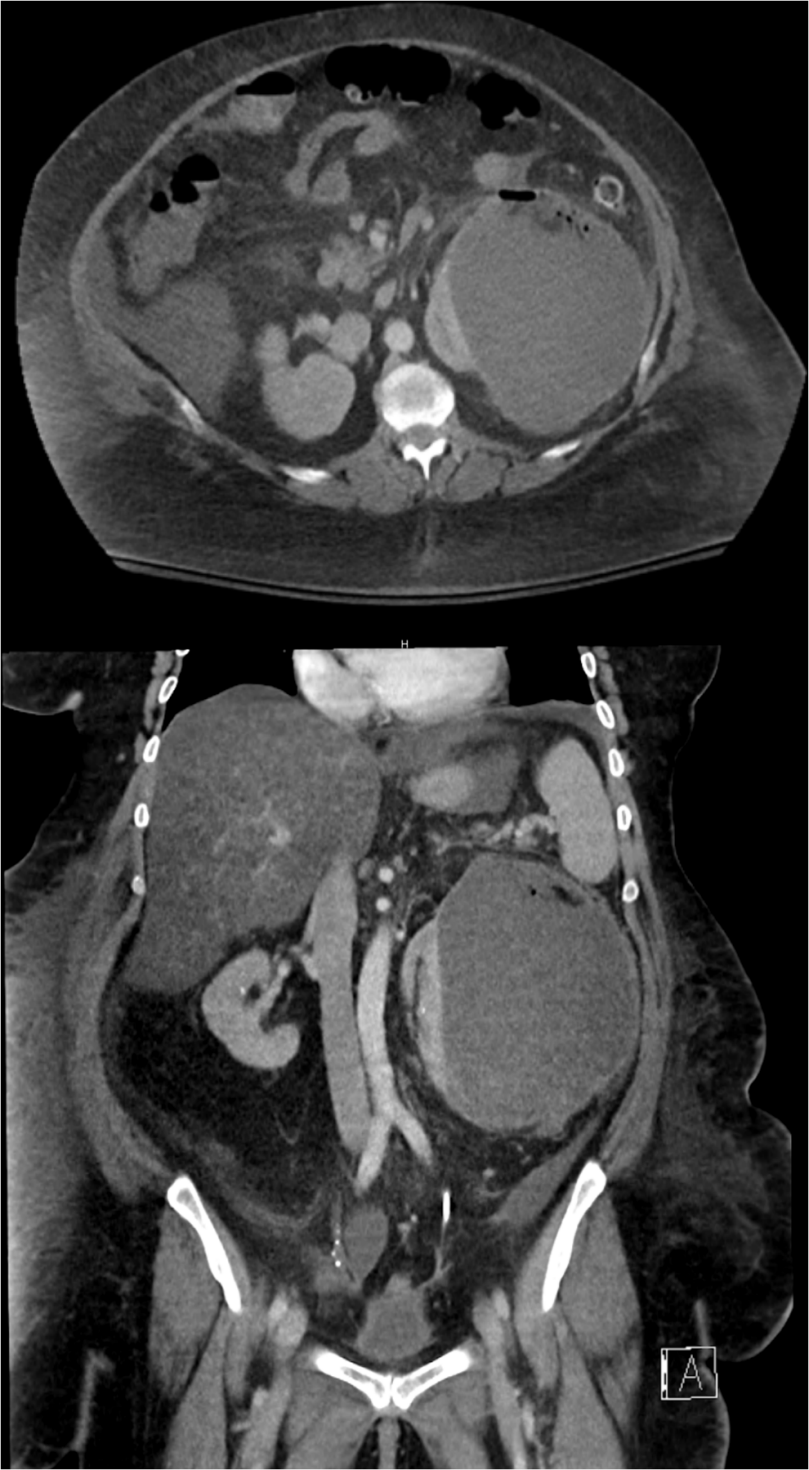
CT scan showing large left perinephric fluid collection containing locules of gas with surrounding inflammatory perinephric fat stranding.

**FIG. 2.**
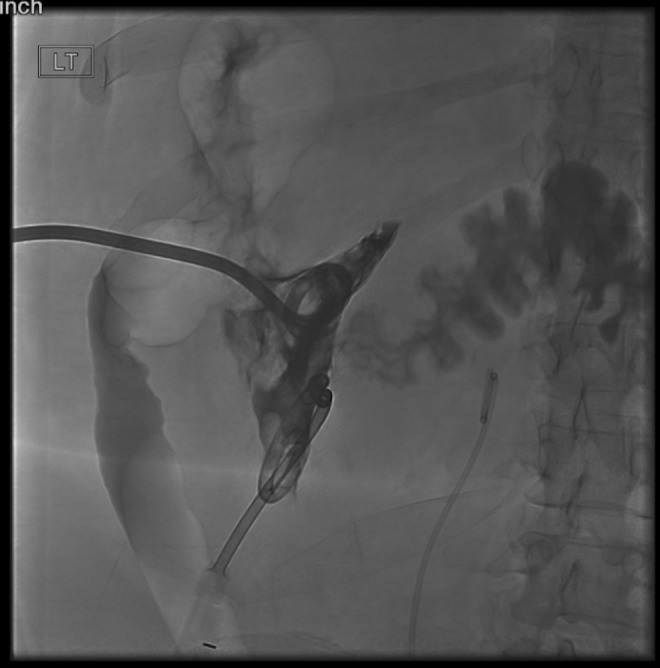
Interventional radiology drain placement showing a fistula from the left subcapsular renal abscess to the proximal descending colon.

In the ED, the patient had signs consistent with severe pyelonephritis. On arrival, her blood pressure was 105/57 mm Hg, heart rate was 97 beats/min, oxygenation was 100% on room air, and temperature was 100.2°F. Laboratory values included creatinine of 1.7 mg/dL (baseline of 1.0 mg/dL), leukocytosis at white blood cell count of 13.1 × 10^3^/mm^3^, anemia at hemoglobin of 9.3 g/dL, and hematocrit of 28%. Her urinalysis was significant for leukocyte esterase, large amount of red blood cells, and bacteria. Despite fluid resuscitation, her blood pressure continued to trend down and she was given vasopressors before being admitted to the intensive care unit on intravenous antibiotics. A repeat CT abdomen and pelvis with contrast showed again the left subscapular hematoma measuring 15.6 × 12.2 × 17.5 cm containing locules of gas with surrounding inflammatory perinephric fat stranding (Fig. 1). At this point, she was taken to interventional radiology for drainage of the presumed large hematoma. During initial puncture, there was 300 mL of frank pus that drained. Fluoroscopic imaging during interventional radiology (IR) drainage revealed a fistula from the left subcapsular abscess to the proximal descending colon (Fig. 2). Drain cultures ultimately confirmed a superimposed polymicrobial infection.

The patient was determined to be an extremely poor surgical candidate owing to her comorbidities; she had an 82% perioperative mortality risk from Child–Pugh class C nonalcoholic steatohepatitis complicated by coagulopathy.^11^ Extensive counseling on the risks of nephrectomy and colectomy was provided to the patient over the course of a few days, and palliative management was offered as well. The patient elected to optimize her nutritional and functional status before proceeding with surgery in an effort to improve her perioperative morbidities. The patient was then discharged to a skilled nursing facility 1 month after the index admission with continued intravenous antibiotics. She continued to decline over the next month with recurrent sepsis and cirrhosis-related complications and unfortunately passed away.

## Discussion and Literature Review

Complication rates for the development of a perinephric hematoma after ureteroscopy are low, cited as 0.45% according to a recently published meta-analysis.^12^ This analysis showed that risk factors include hydronephrosis, preoperative urinary tract infection, hypertension, and a prolonged operative time.^2,13^ Additional factors include female gender, intraoperative ureteral access sheath use, and postoperative ureteral stenting.^13^ The patient described earlier had every one of these risk factors; furthermore, she had severe and decompensated cirrhotic nonalcoholic steatohepatitis with underlying coagulopathy, which greatly increased her risk for the development of a hematoma.

Enterorenal fistulas are extremely rare complications especially after ureteroscopic lithotripsy. Commonly known risk factors for fistula formation include poor nutritional status, recurrent urinary tract infections, sepsis, renal failure or diabetes, and immunosuppression.^14^ Our patient's history of diabetes as well as poor nutritional status with an albumin level of 1.6 g/dL may have predisposed her to fistula formation because of poor healing and lack of sufficient response to infection. Her recurrent urinary tract infections and pyelonephritis likely also contributed to fistula formation. In addition, our patient had additional risk factors for fistula formation, including a prolonged indwelling ureteral stent for 6 months.^15^ Interestingly, the patient had a history of radiation therapy for uterine malignancy about 10 years prior, which had caused her to develop radiation enteritis and recurrent colonic bleeding. It is well known that radiation injury may lead to the formation of fistula, abscess, and strictures.^16^ Finally, the patient's repeated sessions of ureteroscopic lithotripsy with use of ureteral access sheath with prolonged operative times may have contributed to her increased risk of fistula formation.^17^

Despite her large stone burden, she underwent an unusually high number of lithotripsies after declining a percutaneous nephrolithotomy. A study published in 2011 reported 100% stone-free rate within just two ureteroscopic sessions for stones measuring 2 to 4 cm.^18^ Wheat et al. described a mean number of 2.3 procedures for obese patients with mean stone diameter of 3.8 cm.^19^ Our patient also had a 3.8 cm stone but had required five procedures, an excessive number of sessions because of her large stone burden, and also had developed Steinstrasse formation despite stent placement.

With any kind of enterorenal fistula, copious diarrhea has been described in the literature as a primary symptom, which our patient reported on admission.^20^ Her fistula was found incidentally during IR drain placement. Having a high index of suspicion for this rare diagnosis is warranted, specifically in the setting of a highly morbid patient with numerous kidney interventions and a history of radiation enteritis.

The patient presented had a complicated medical history with multiple comorbidities that made it difficult to pursue a surgical course owing to her high risk of perioperative mortality.

## Conclusion

A subscapular hematoma evolving into a perinephric abscess is a rare but known complication of ureteroscopic lithotripsy; however, this patient developed an enterorenal fistula that has yet to be reported after repeated ureteroscopy.

## Abbreviations Used

CT: computed tomography
ED: emergency department
IR: interventional radiology
